# Health workers’ knowledge of and attitudes towards computer applications in rural African health facilities

**DOI:** 10.3402/gha.v7.24534

**Published:** 2014-10-27

**Authors:** Felix Sukums, Nathan Mensah, Rose Mpembeni, Jens Kaltschmidt, Walter E. Haefeli, Antje Blank

**Affiliations:** 1 Department of Clinical Pharmacology & Pharmacoepidemiology, Heidelberg University Hospital, Heidelberg, Germany; 2 Directorate of Information and Communication Technology, Muhimbili University of Health and Allied Sciences (MUHAS), Dar es Salaam, Tanzania; 3 Navrongo Health Research Centre, Navrongo, Ghana; 4 Department of Epidemiology and Biostatistics, School of Public Health and Social Sciences, Muhimbili University of Health and Allied Sciences (MUHAS), Dar es Salaam, Tanzania

**Keywords:** computers, computer knowledge, attitude towards computers, rural health services, health personnel, maternal health services, sub-Saharan Africa

## Abstract

**Background:**

The QUALMAT (Quality of Maternal and Prenatal Care: Bridging the Know-do Gap) project has introduced an electronic clinical decision support system (CDSS) for pre-natal and maternal care services in rural primary health facilities in Burkina Faso, Ghana, and Tanzania.

**Objective:**

To report an assessment of health providers’ computer knowledge, experience, and attitudes prior to the implementation of the QUALMAT electronic CDSS.

**Design:**

A cross-sectional study was conducted with providers in 24 QUALMAT project sites. Information was collected using structured questionnaires. Chi-squared tests and one-way ANOVA describe the association between computer knowledge, attitudes, and other factors. Semi-structured interviews and focus groups were conducted to gain further insights.

**Results:**

A total of 108 providers responded, 63% were from Tanzania and 37% from Ghana. The mean age was 37.6 years, and 79% were female. Only 40% had ever used computers, and 29% had prior computer training. About 80% were computer illiterate or beginners. Educational level, age, and years of work experience were significantly associated with computer knowledge (*p*<0.01). Most (95.3%) had positive attitudes towards computers – average score (±SD) of 37.2 (±4.9). Females had significantly lower scores than males. Interviews and group discussions showed that although most were lacking computer knowledge and experience, they were optimistic about overcoming challenges associated with the introduction of computers in their workplace.

**Conclusions:**

Given the low levels of computer knowledge among rural health workers in Africa, it is important to provide adequate training and support to ensure the successful uptake of electronic CDSSs in these settings. The positive attitudes to computers found in this study underscore that also rural care providers are ready to use such technology.

The implementation of information and communication technologies (ICT) can positively influence many aspects of healthcare provision (1–4). Among health information technology (HIT) applications, the use of computerized provider order entry (CPOE) linked to computerized clinical decision support systems (CDSSs) is the most promising approach. These applications improved patient care through data processing and the provision of patient-specific evidence, and also through health workers’ increased adherence to clinical practice guidelines (2, 3, 5–8).

The implementation of HIT applications in developing countries has been slow, particularly in rural areas (4, 9, 10). Several issues, such as technical infrastructure, and computer knowledge, skills, experience, and attitudes of prospective users, need to be addressed (11–14) before introducing computers in rural healthcare settings (11, 15, 16). There is evidence that health workers’ interest, knowledge and skills of computer applications can influence their acceptance, or not, of HIT solutions in the workplace (15, 17–19). Positive attitudes are important (20, 21) and the willingness of health workers to use any HIT system is influenced by their perceptions of its value, clinical benefits, and ease of use (14, 22–26).

In developing countries the successful adoption of HIT is hindered by, among other things, insufficient technical infrastructure (12, 27, 28). Where this is due to the non-existence or lack of reliable electricity, solar power can offer an alternative (29, 30). Moreover, adverse attitudes and inadequate computer knowledge and skills among healthcare workers can also negatively impact on the adoption of computer systems (9, 15, 31, 32). In rural India, the implementation of a CDSS increased patient visits, but the increased documentation workload impacted negatively on health workers’ attitudes (10). Not only do many rural health workers in developing countries have limited exposure to computer applications, but due to lack of IT infrastructure and support, they are often also sceptical about the adoption of computers in their workplace (9, 30). Inadequate training and support and limited computer access are some of the well-known reasons for pessimistic attitudes (33).

Currently the literature lacks information about the knowledge and attitudes of health workers towards computer applications in primary healthcare facilities in rural African settings. Any assessment of the issue is complicated because many health workers have never been exposed to computers and therefore have difficulty conceptualising the possible benefits of a proposed CDSS.

This paper refers to a study conducted in Ghana and Tanzania as a sub-study of the larger QUALMAT research project. The QUALMAT project (Quality of Maternal and Prenatal Care: Bridging the Know-do Gap) was funded as part of the 7th Framework Programme of the European Union (grant agreement 22982) and is a collaboration between the Centre de Recherche en Santé de Nouna (Burkina Faso), Ghent University (Belgium), Heidelberg University (Germany), Karolinska Institutet (Sweden), Muhimbili University of Health and Allied Sciences (Tanzania), and Navrongo Health Research Centre (Ghana). The overall objective of the QUALMAT research is to improve the motivation and performance of health workers and ultimately the quality of pre-natal and maternal care services. The QUALMAT sub-study is an intervention project. This involves the development and implementation of a system of performance-based incentives and a computer-assisted CDSS based on World Health Organization guidelines. All interventions were evaluated using a pre–post controlled study design in rural Burkina Faso, Ghana, and Tanzania between 2009 and 2014 (34).

Prior to the introduction of the CDSS, a baseline assessment was conducted in order to ascertain health workers’ knowledge of and attitudes towards computer applications. This paper presents the findings of the pre-implementation assessment of the computer knowledge, experience, and attitudes of health workers in 24 QUALMAT project sites in Ghana and Tanzania.

## Methods

The study was conducted from September through December 2011 in two resource-poor countries in Africa, namely Ghana and Tanzania. A third country Burkina Faso was part of the QUALMAT study, but was unable to provide data for this sub-study. In each country, six rural primary healthcare facilities from one district served as intervention sites, and six other primary healthcare facilities were selected from the second district to be non-intervention sites for the QUALMAT study. The intervention districts were Kassena-Nankana (Ghana) and Lindi rural (Tanzania) while Builsa and Mtwara were the non-intervention districts in Ghana and Tanzania, respectively. The approximate population size of these districts is 143,000 for Kassena-Nankana, 95,800 for Builsa, 216,000 for Lindi, and 205,000 for Mtwara. The study sites are disadvantaged rural districts selected by the QUALMAT project consortium.

The study population (*N*=115) included all health workers who were involved in the provision of maternal and newborn care at the study sites and comprised medical attendants/nursing assistants, midwives and community nurses, nurses, clinical officers, and assistant medical officers. Staff were grouped into three categories. Nurses and midwives were grouped as nursing staff, clinicians as medical staff, and unskilled and auxiliary/support staff as ‘other’ staff.

A questionnaire was developed to collect socio-demographic information such as age, sex, and professional qualifications of the health worker including educational level, job title, years of work experience in maternal and newborn care, workplace, and previous computer training and use (if any). Self-rated computer knowledge was measured using three categories: illiterate/do not have knowledge, beginner/low knowledge, and basic/good knowledge (Tables 1 and 2). The questionnaire included nine computer attitude statements that were rated on a five-point Likert scale ranging from ‘1’ (strongly disagree) to ‘5’ (strongly agree). The statements were based on the health workers’ perception of the application of computers in their work, perceived benefits, and their willingness and ability to use a computer during patient care. The scale was informed by published literature (11, 13, 15, 20, 35) taking the rural African settings into account (12, 32, 36). Among nine computer attitude statements (Table 3), four were negatively and five positively phrased. The order of appearance was randomized in order to reduce response bias (20).

**Table 1:** Socio-demographic characteristics of the respondents in QUALMAT sites in Tanzania and Ghana, December 2011 (*N*=108)

Variable	Respondents, *N* (%)
District name	
Lindi (Tanzania)	40 (37.0)
Mtwara (Tanzania)	28 (26.0)
Kassena-Nankana (Ghana)	21 (19.4)
Builsa (Ghana)	19 (17.6)
Sex	
Female	85 (78.7)
Male	23 (21.3)
Age group	
20–34	48 (44.5)
35–49	40 (37.0)
50–64	20 (18.5)
Educational level	
Primary and secondary school	27 (25.0)
College certificate	58 (53.7)
College diploma	23 (21.3)
Cadre	
Medical staff (clinicians)	13 (12.0)
Nursing staff	67 (62.0)
Other staff	28 (26.0)
Experience in maternal and child healthcare (years)	
1–5	45 (41.7)
6–10	16 (14.8)
11 or above	47 (43.5)
Total	108 (100)

**Table 2:** Computer knowledge and experience among respondents in the QUALMAT sites in Tanzania and Ghana, December 2011 (*N*=108)

	Respondents, *N* (%)	
	
Attribute	Tanzania	Ghana
Computer training?		
No	52 (76.5)	25 (62.5)
Yes	16 (23.5)	15 (37.5)
Computer program studied (*N*=31)^a^		
Introduction to computers	16 (29.1)	10 (35.7)
Microsoft Word	8 (15.7)	10 (35.7)
Microsoft Excel	5 (10.2)	4 (14.3)
Microsoft PowerPoint	4 (8.2)	2 (7.1)
Internet and email	3 (6.1)	4 (14.3)
Microsoft Access	1 (1.5)	0 (0.0)
Computer knowledge (*N*=104)		
Illiterate	43 (63.2)	2 (5.6)
Beginner	17 (25.0)	22 (61.1)
Basic user	8 (11.8)	12 (33.3)
Ever used computers?		
No	48 (70.6)	16 (40.0)
Yes	20 (29.4)	24 (60.0)
Place of computer use (*N*=44)^a^		
At home	8 (42.1)	5 (20.8)
At work	3 (15.8)	13 (54.2)
Internet café	2 (10.5)	5 (20.8)
Other	7 (31.6)	1 (4.2)
Using mobile phone to access Internet services		
No	62 (91.2)	27 (67.5)
Yes	6 (8.8)	13 (32.5)

a Multiple answers possible.

**Table 3:** Mean self-ratings of computer attitudes among respondents in QUALMAT sites in Tanzania and Ghana, December 2011 (*N*=106)^a^

No.	Statement	Mean^b^	SD
1	I believe computers/computerized clinical decision support system (CDSS) can assist me in my work at the health centre	4.58	0.63
2	I will be capable to use the computer/CDSS during patient care	3.96	0.93
3	I think using computers will increase workload in duties in my health centre	2.37	1.24
4	I believe computers can support my clinical decision making during provision of healthcare	4.08	1.04
5	I will not be capable to learn and use CDSS and other computer systems	1.81	0.94
6	I think use of CDSS will certainly improve quality of care	4.39	0.84
7	I do not have time to learn CDSS and other computer systems	1.84	0.94
8	I do not have time to use CDSS and other computer systems	1.93	1.02
9	I believe health workers in my centre are willing to learn/use computers at their workplace	4.38	0.94

Answers were offered on a Likert type scale where ‘1’ indicated ‘strongly disagree’, and ‘5’ indicated ‘strongly agree’.

a *N* does not add to 108 due to missing values which were dropped pairwise.

b These are means of each attitude statement as opposed to the composite attitudes scores in Table 5.

The questionnaire was prepared in English and translated to Kiswahili for use in Tanzania. The questionnaires were pretested in rural health facilities in Coast region, Tanzania. Four research assistants were trained in Tanzania to support the local researcher (FS) and collect data using the questionnaire, while in Ghana, the researcher (NM) and one research assistant collected the data. The health workers were informed and interviewed at their workplace. In the intervention district, health workers who were not at their work stations on the day of the interview (e.g. because they were on annual leave) were interviewed during the computer training (only in Tanzania). In the non-intervention district call backs were made to ensure participation by as many as possible.

Quantitative findings from the questionnaires were augmented with findings from semi-structured interviews and focus group discussions. The aim was to better understand health workers’ previous computer experience, any training received, including when and how it occurred, as well as their knowledge, attitudes, and expectations about the forthcoming computer system. The questions asked in the interviews and group discussions were derived from the questionnaire and interviewers probed to generate discussion. Participants were also asked to comment on their preparedness and what they anticipated as the possible benefits.

The structured questionnaires were completed prior to the interviews and discussions. A convenience sample of eight healthcare workers at the study sites in Lindi (intervention) and Mtwara (non-intervention) was purposively selected for participation in semi-structured one-on-one interviews. The interviewer (FS) audio-taped the interviews and took field notes. The focus group discussions were conducted prior to the computer training sessions only in Lindi (intervention district). A total of 39 health workers participated in the focus groups. The participants were randomly assigned by the district offices for participation in the computer training depending on their convenience and work schedules. The number of people interviewed and the duration of the group discussions were determined by the researcher. The interviews and the group discussions were stopped when the researcher no longer received new information related to the research question (data saturation). Three facilitators including the investigator (FS) took notes of issues raised during the group discussions. At the end of each day, the notes were reviewed and discussed to identify and interpret the respondents’ computer experiences and perceptions of computer applications. The investigator then compiled the notes into a single report which was used for analysis. The feedback from the respondents during the discussions was also used to inform and revise the training plan in order to provide more insights and explanation especially regarding any preconceptions and fears that were presented.

The study was approved by the Ethics Committee of the Medical Faculty of the University of Heidelberg, Germany (S-173/2008), the Institutional Review Board at the Navrongo Health Research Centre, Ghana (ID: NHRCIRB116), and the Muhimbili University of Health and Allied Sciences Ethical Review Committee, Tanzania (Ref. No.MU/RP/AEC/Vo.XIII/). In addition, the district authorities granted permission to conduct the study. Information sheets describing the objectives of the study and the anonymous questionnaire were distributed. Participation in the study was voluntary. All participants gave their written consent. They also had the right to withdraw from the study at any time without undue consequences.

### Data analysis

The quantitative data were analysed using SPSS version 17 (SPSS Inc., Chicago, IL). Descriptive statistics included frequency distributions and measure of central tendency and variability. Chi-squared tests (*X* ^2^) were used to examine associations between the computer knowledge level and selected socio-demographic characteristics. Scores on questions which asked about attitudes were combined into summary scores (20). The reliability of the attitudes scale was assessed using Cronbach's alpha coefficient.

One-way analysis of variance (F) and Tukey's-b post hoc test were used to identify the association between health workers’ computer attitudes and selected variables. The results are reported as mean±standard deviation (SD), or 95% confidence intervals (CI). All statistical values were considered significant at the *p*≤0.05.

Analysis of qualitative data was undertaken simultaneously with the questionnaire data in order to explore relevant issues in the focus groups and interviews (37). The qualitative data were investigated using thematic analysis. Interview transcripts and discussion reports were retrieved and entered into a text file (38). The text file was thoroughly read to develop codes and themes in line with the questionnaire topics (computer knowledge, experiences, and attitudes towards computer applications). The results were used to identify major topics discussed. Qualitative data were compared to the questionnaire results, and similarities and differences were identified. The respondents’ views and some quotes were selected to enrich the quantitative findings.

## Results

### Socio-demographic characteristics of the respondents

From 115 targeted health workers, 108 (94% response rate) responded to the questionnaire (Table 1) including 68 (63%) from Tanzania and 40 (37%) from Ghana. All health workers from Lindi, Builsa, and Kassena-Nankana participated in this study while seven health workers in Mtwara did not because they were not available during the data collection. The mean age (±SD) was 37.6 (±11.0) ranging from 21 to 59 years. The mean number of years of work experience in maternal and newborn care was 10.5 (±9.3). Eight participants were interviewed for about 10 min each. Three discussion group sessions of, on average, 15 min each were held in Lindi, Tanzania. The group discussion sessions were attended by 9–15 participants. In total 39 participated.

### Computer knowledge, experience and usage

Table 2 indicates computer knowledge and experience among the health workers working in rural health facilities of the four districts. Thirty-one (28.7%) reported that they had prior computer training. Training topics were diverse and included the introduction to computers, training for software such as Microsoft Word, Microsoft Excel and also Internet or email training. The duration of previous computer training ranged from 1 day to 2 years.

During the interviews and discussions, the participants provided details about their previous computer training and experience (if any) and their expectations about using computers in their work. Some of the respondents attended computer training for only one computer program, for example, Microsoft Word, while others reported to have studied up to six different programs. Some were unable to provide details of the software they had used. Many reported limited hands-on experience. Some respondents reported having received some earlier computer training (e.g., up to 7 years ago) but they had rarely used computer systems since that time and had forgotten most of what they had learnt. Only a small number (four) were able to explain hardware and software functions.

Sixty-four (59.3%) of questionnaire respondents indicated that they had never used computers prior to the QUALMAT project having been introduced at their sites. Only 45 (43.3%) respondents self-assessed as being computer illiterate in the questionnaire. Some respondents had only been briefly exposed to computers during the introduction of the project. Nineteen (17.6%) indicated that they had used mobile phones for Internet and email services of which only 10 (52.6%) had computer training.

Table 4 shows a statistically significant difference in computer knowledge between staff groups. More nurses (25.4%) rated their computer knowledge level as basic than medical (7.6%) and other staff (10.7%). Conversely, most members of the other staff group (82.1%) were computer illiterate (*N*=23). In contrast to educational level, sex was not associated with computer knowledge. Only 26.1% of the people with a college diploma were computer illiterate compared with 88.9% of unskilled health workers, of which only one indicated a basic computer knowledge level.

**Table 4:** Association between computer knowledge and other attributes the respondents in QUALMAT sites in Tanzania and Ghana, December 2011 (*N*=104)

	Computer knowledge (*N*=104)^a^				
			
Variable (% female)	Illiterate (%), *N*=45	Beginner (%), *N*=39	Basic user (%), *N*=20	*X* ^2^	*p*
Sex					
Female	33 (40.7)	33 (40.7)	15 (18.6)	1.6	0.43
Male	12 (52.2)	6 (26.1)	5 (21.7)		
Age group (years)					
20–34 (52.9)	10 (21.3)	22 (46.8)	15 (31.9)		
35–49 (31.8)	22 (57.9)	11 (28.9)	5 (13.2)	20.2	<0.01
50–64 (15.3)	13 (68.4)	6 (31.6)	0 (0)		
District					
Lindi, Tanzania (32.9)	27 (67.5)	9 (22.5)	4 (10.0)		
Mtwara, Tanzania (21.2)	16 (57.1)	8 (28.6)	4 (14.3)	33.7	<0.01
Kassena-Nankana, Ghana (24.7)	1 (4.8)	14 (66.7)	6 (28.6)		
Builsa, Ghana (21.2)	1 (6.7)	8 (53.3)	6 (40.0)		
Country					
Tanzania (54.1)	43 (63.2)	17 (25)	8 (11.8)	32.2	<0.01
Ghana (45.9)	2 (5.6)	22 (61.1)	12 (33.3)		
Education level					
Unskilled: primary/secondary school (25.9)	24 (88.9)	2 (7.4)	1 (3.7)		
College certificate (61.2)	15 (27.8)	26 (48.1)	13 (24.1)	30.9	<0.01
College diploma (12.9)	6 (26.1)	11 (47.8)	6 (26.1)		
Experience in maternal and child healthcare (years)					
5 or below (45.9)	8 (17.8)	21 (46.7)	16 (35.6)		
6–10 (15.3)	6 (46.2)	5 (38.5)	2 (15.4)	26.6	<0.01
11 or above (38.8)	31 (67.4)	13 (28.3)	2 (4.3)		
Job title/cadre					
Medical staff (2.4)	6 (46.2)	6 (46.2)	1 (7.6)		
Nursing staff (72.9)	16 (25.4)	31 (49.2)	16 (25.4)	27.1	<0.01
Other staff (24.7)	23 (82.1)	2 (7.2)	3 (10.7)		
Computer usage prior to this study					
Yes (42.4)	0 (0.0)	24 (54.5)	20 (45.5)	66.2	<0.01
No (57.6)	45 (75.0)	14 (25)	0 (0.0)		
Computer training attendance^b^					
Yes (29.4)	0 (0.0)	17 (54.8)	14 (45.2)	38.1	<0.01
No (70.6)	45 (61.6)	22 (30.1)	6 (8.2)		

a *N* may not add to 108 due to missing values which were dropped pairwise.

b Total may not add to 100% due to rounding.

Age was inversely correlated with computer literacy, as were years of experience in maternal and child health (MCH). Respondents with five or less years, that is, younger people, more often reported basic computer knowledge. In contrast, the majority of respondents with 11 or more years of professional experience (67.4%) were computer illiterate followed by those with 6–10 years of MCH experience (46.2%, Table 4). During interviews and discussions, younger providers expressed confidence in their ability to swiftly learn and use computers despite their having had limited prior exposure. Some older providers were pessimistic about being able to quickly learn and use computers. For example, one provider stated, ‘Younger care providers are going to benefit a lot from computer use as they are fast to learn and use the technology. Look for examples even with use of phones they already have access to Internet (social media) while I use mine for calling and message texting only’ (Male clinical officer). Possibly reflecting the fact that in Ghana health workers had been exposed to computers through various health projects, computer illiteracy differed markedly between the two countries. Further the fraction of staff members with basic computer skills in Ghana was ~3 times larger than in Tanzania. Computer experience was correlated with computer knowledge whereby a quarter of respondents without previous computer experience rated themselves as beginners while 10 (45.5%) of respondents with computer experience were rated at the basic level. Those who had previously used computers were more confident about working with computers in the future.

We did not explore the frequency of computer use in the questionnaire. However, during interviews and discussions most of the healthcare workers indicated that they did not have access to computers in their work place. In Tanzania, no facility had computers. During the discussions, access to computer training and the use of computers at the work place was questioned. Fears were expressed that the lack of staff, computers, funding for training, and time to practice may negatively impact the planned intervention. However, computers were eagerly awaited and as one provider said, ‘At last I have got a chance to attend computer training, we made several requests to the district office for computer training but there were no funds for and time to attend the training’ (Male clinical officer).

### Health workers’ attitudes towards computer application

Reliability tests of the attitude scale resulted in a good Cronbach score of 0.78. Health workers’ attitudes towards computer applications in healthcare are presented as the total score (20). The mean (±SD) attitude score was 37.2 (±4.9) with a range of 27 (18–45). Almost all health workers (95.3%) had a mean score of above 27 (those who selected neutral (3) for all nine attitude statements). On average, female health workers had a lower mean score on attitudes towards computers (36.6±5.1) compared to males (39.4±4.0, *p*<0.05) and health workers from Tanzania had slightly higher mean scores (38.5±4.5) compared to those from Ghana (34.9±5.1, *p*<0.01).

The mean scores were: 37.4±3.9 for unskilled health workers, 36.3±5.7 for those with a college certificate, and 39.6±3.1 for college diploma holders. The higher score among those with diploma level education compared with other groups was statistically significant (*F*=3.79, *p*<0.05). There was no significant difference in the mean attitudes score in relation to health workers’ age, cadre, computer knowledge, computer usage, and years of experience in MCH as shown in Table 5. During interviews and discussions, most of the providers expressed positive attitudes towards computers in their workplace. But some, especially older nurses, were not confident in their ability to swiftly learn and cope with the use of new technology at work. Those who were going to retire in a few years did not anticipate great benefits. When asked about the possible advantages of computers at their workplace most respondents expected benefits deriving from administrative support, such as assistance in record keeping and preparation of reports. ‘Computer will simplify my work’, ‘It will help in record keeping’, or ‘It will improve patient care’ were the most frequently stated answers. One provider emphasized another aspect, ‘We will be seen by our colleagues as well as patients as modern care providers when using this technology during patient care’ (Female nurse midwife). Two respondents expressed disappointment that they would not be able to benefit from the technology change because of the forthcoming transfers from the study sites. Expressing excitement and willingness to learn and use computer one provider stated, ‘I never thought in my life I will get the privilege to learn (how to use) computers, I am very grateful for this opportunity’ (Medical attendant, Lindi). During group discussions, the participants also expressed their concerns about the need for extensive training and the time required for building capacity in computer use in relation to patient care. They also feared that huge workloads, inadequate staffing, and insufficient infrastructure may hinder successful implementation. Doubts were also expressed regarding the availability of sufficient local technical support at the facilities.

**Table 5:** Attitudes towards computers among respondents in QUALMAT sites in Tanzania and Ghana, December 2011 (*N*=106)^a^

			ANOVA	
			
Variable (% female)	*N* (%)	Attitude (total score) Mean±SD (95% CI)^b^	F	*p*
Sex				
Male	23 (21.7)	39.4±4.0 (35.5–37.7)	5.89	0.017
Female	83 (78.3)	36.6±5.1 (37.7–41.1)		
Age (years)				
20–34 (52.9)	48 (45.3)	36.5±5.6 (34.9–38.1)		
35–49 (31.8)	39 (36.8)	38.6±3.3 (37.5–38.6)	2.37	0.099
50–64 (15.3)	19 (17.9)	36.2±5.9 (33.4–39.1)		
District^c^				
Lindi, Tanzania (32.9)	40 (37.7)	38.2±5.0 (36.6–39.8)		
Mtwara, Tanzania (21.2)	28 (26.4)	38.9±3.6 (37.6–40.3)	4.96	0.003
Kassena-Nankana, Ghana (24.7)	21 (19.8)	35.3±4.6 (33.2–37.4)		
Builsa, Ghana (21.2)	17 (16.0)	34.4±5.7 (31.5–37.4)		
Country				
Tanzania (54.1)	68 (64.2)	38.5±4.5 (37.4–39.6)	14.4	<0.001
Ghana (45.9)	38 (35.8)	34.9±5.1 (33.2–36.6)		
Education level^c^				
Unskilled: primary/secondary school (25.9)	27 (25.5)	37.4±3.9 (35.9–39.0)		
College certificate (61.2)	57 (23.8)	36.3±5.7 (34.7–37.8)	3.79	0.026
College diploma (12.9)	22 (20.7)	39.6±3.1 (38.2–40.9)^d^		
Experience in maternal and child healthcare (years)				
5 or below (45.9)	45 (42.5)	36.6±5.5 (35.0–38.3)		
6–10 (15.3)	15 (14.2)	37.7±4.2 (35.4–40.0)	0.563	0.572
11 or above (38.8)	46 (43.4)	37.7±4.7 (36.3–39.1)		
Job title/cadre				
Medical staff (2.4)	13 (12.3)	39.9±3.5 (37.8–42.1)		
Nursing staff (72.9)	65 (63.3)	36.5±5.5 (35.2–37.9)	2.64	0.076
Other staff (24.7)	28 (26.4)	37.5±3.9 (35.9–39.1)		
Computer knowledge				
Illiterate (40.7)	45 (43.7)	37.7±4.9 (36.2–39.2)		
Beginner (40.7)	38 (36.9)	39.2±5.5 (35.2–38.7)	0.24	0.785
Basic (18.5)	20 (19.4)	37.0±4.5 (34.9–39.1)		
Computer usage prior to this study				
Yes (42.4)	42 (40.6)	35.3±5.3 (35.3–38.6)	0.22	0.638
No (57.6)	63 (59.4)	37.4±4.8 (36.2–38.6)		

a *N* does not add to 108 due to missing values which were dropped pairwise.

b Mean total attitudes score indicates summarized score of all nine items (higher score represents more positive attitudes towards computers; Mean total score is 37.2±4.9).

c Total may not add to 100% due to rounding.

d Significant difference vs. both other groups.

## Discussion

Requirements for the success of computer-based interventions in healthcare include the familiarity of the users with the technology (9, 35) and positive mental attitudes and motivation regarding computer use (11, 35, 39). The results of our survey suggest that the majority of the health workers in these rural facilities had none or very limited training for and exposure to computer applications. Respondents from Ghana reported better knowledge and exposure to computers compared to Tanzania, reflecting the fact that in Ghana health workers had been exposed through various health projects such as the introduction of computers for health insurance purposes.

Similar studies in sub-Saharan Africa have also reported low computer knowledge among health workers (36, 39, 40). In our study, 77 health workers (71.3%) had no previous computer training and only 40.7% had previously used computers, a figure that is substantially lower than, for example, the findings in Ireland 10 years ago where 63.5% of nursing students had had computer training in a baseline assessment (41). In our study even those who indicated having attended computer training reported variable duration and coverage of topics. Although it is difficult to draw comparisons, this suggests that health workers in rural primary healthcare facilities in sub-Saharan Africa have been lagging behind their colleagues in the developed world by more than a decade.

Studies conducted in urban settings in developing countries have also showed that health workers have limited access to computer training and have low levels of computer knowledge. A study conducted in city hospitals of Addis Ababa in Ethiopia reported that 49.6% of the health workers had previous computer training, but 80% of health workers rated themselves as using computers inadequately (33). In another study, conducted in Kenyatta National Hospital in Nairobi Kenya, at least 64.5% had previous computer training, but 40.1% of nurse managers did not have access to computers (36). These problems are even worse in rural settings. At the QUALMAT project sites only 23.5 and 37.5% had had computer training, and 88.2 and 66.7%, respectively, in Tanzania and Ghana reported that they were computer illiterate or beginners. Furthermore, the majority of the providers did not have access to computers. In Tanzania, no facility had a computer. These results indicate a digital divide between healthcare facilities in urban and rural areas of the sub-Saharan countries. This is certainly due in part to the difficulties of maintaining reliable electricity supplies in rural African settings.

It is important to note that computer knowledge was self-reported, reflecting health workers’ confidence, understanding, and experience rather than objectively quantifying their actual knowledge and skills. The quantitative findings, however, were also supported by the results of the interviews and focus group discussions in which most of the participants were unable to demonstrate understanding of basic hardware and software.

Furthermore, although self-rated competency is not equivalent to objective levels of computer knowledge and skills for using computers, it is a major and important indicator of confidence and ability prior to training (42). It may, on a small scale, therefore help to guide training sessions, allow ‘expert’ users to be identified, and indicate where individual coaching is needed (43). In this study, health workers’ age, cadre, and educational level were significantly associated with computer knowledge. This contrasts with a study conducted in Nigeria in which health professionals’ age, rank, and sex did not influence their computer knowledge, attitudes, or utilization patterns (39). However, it is difficult to make direct comparisons because the Nigerian study included only well-educated physicians, medical students, and health record officers.

It is generally accepted that the HIT sector will undergo accelerated development in African countries. A major challenge is how to best involve the rural areas of resource-poor countries in this development. In Ghana and Tanzania, the responsible ministries/agencies have introduced basic computer application courses in the medical and nursing curriculum, and health training institutions are now implementing basic computer training modules and some have computer laboratories. These efforts may well build capacity for the implementation of computer systems (9). It may be important for computer-related training to become a routine part of the continuous education and professional development of health workers in rural areas in order to facilitate the successful adoption of HIT.

Consistent with other studies (15, 31, 36), this study shows that an overwhelming majority of health workers (95.3%) had positive attitudes towards computer applications in healthcare and that they expected that computers could potentially improve patient care and simplify their work. Male health workers had higher attitude scores which was contrary to what was seen in Kuwait where female nurses had more positive attitudes to computers (31). No significant association was observed between computer knowledge, training, usage, and attitudes in the present study. A study conducted in India (15) showed that nurses’ computer attitudes were influenced by age and sex and not by the amount of computer usage. The positive attitude of the often computer illiterate health workers may be attributed to their readiness to learn and use computers. It is important that new users are supported, encouraged, and followed up, after exposure to computers in the healthcare workplace (44, 45). A study conducted among nurses in Kuwait showed that attitudes towards computerization were associated with educational level, computer experience, and duration of computer use whereby female respondents and those with a bachelor's degree and some computer experience indicated more positive attitudes. The predictors of attitudes in that study were nationality, sex, and duration of computer use (31).

In this study, health workers in Tanzania on average scored higher on computer attitudes and health workers in Ghana were significantly more computer literate. There are factors that may explain this difference. Health workers in Tanzania had less exposure to computers, and sites in Ghana have already experienced a greater penetration of computers and software systems due to the introduction of the electronic health insurance system. But the exposure has apparently depressed enthusiasm among those health workers in Ghana. This underscores nicely the importance of promoting and supporting attitudes after the introduction of computer systems.

A follow-up study will be conducted to determine contributing factors to changes in computer attitudes after the introduction of the QUALMAT CDSS.

### Study limitations

This study has a number of limitations. We did not attempt to objectively assess computer skills and relate them to the subjective self-assessment of the interviewees. However, the large proportion of respondents who reported very little computer knowledge and limited access to computers suggests a lack of necessary skills.

Another limitation is the relatively small sample size of facilities and health workers included in this survey. A larger sample size would have provided a greater statistical power. Because this is a sub-study of the QUALMAT project, the sample size was restricted by the number of personnel at the QUALMAT study sites and the capacity of the sites to conduct the study in a patient care setting. Personnel were sometimes involved in parallel study activities. Therefore, qualitative data collection could, for example, not be carried out in Ghana. However, we emphasize the excellent response rate of 94% for the questionnaires. In addition, data were collected in four different rural districts within two countries with considerable concordance of findings suggesting that these results may be generalizable to other similar rural resource-poor settings. In addition, qualitative methods complemented the quantitative data. The qualitative data supported the quantitative findings and provided some insights into the experiences and perceptions of the health workers.

## Conclusions and recommendation

Health workers’ computer knowledge and attitudes have an important bearing on the uptake and utilization of computer systems in the workplace. In this study, most health workers in remote rural African primary health facilities had little computer knowledge, yet they had positive attitudes and expressed willingness to adopt the technology. These results are promising for the introduction of technical developments such as the QUALMAT CDSS.
